# Application of Deep Brain Stimulation in Refractory Post-Traumatic Stress Disorder

**DOI:** 10.7759/cureus.33780

**Published:** 2023-01-14

**Authors:** Vainavi Khitha, Surekha Tayade

**Affiliations:** 1 Department of Psychiatry, Jawaharlal Nehru Medical College, Datta Meghe Institute of Medical Sciences (Deemed to be University), Wardha, IND; 2 Department of Obstetrics and Gynaecology, Jawaharlal Nehru Medical College, Datta Meghe Institute of Medical Sciences (Deemed to be University), Wardha, IND

**Keywords:** post traumatic stress disorder (ptsd), ptsd, shell shock syndrome, post traumatic stress disorder, neurostimulation, neuromodulation, deep brain stimulation

## Abstract

Post-traumatic stress disorder (PTSD) is a mental disorder that produces crippling anxiety and occurs in response to an extreme, traumatic stressor. Compared to the prevalence of PTSD in the general population, the prevalence of PTSD in at-risk populations (e.g., army veterans, those affected by environmental calamities, and others) can reach up to threefold. The conventional treatment of PTSD involves using SSRIs (serotonin reuptake inhibitors) and other anti-depressants along with psychotherapy such as debriefing and CBT (cognitive behavioral therapy). Due to increasing resistance to conventional treatment, more novel treatment options, such as stellate ganglion block shots and neuromodulation, are being explored. These neuromodulation techniques include transcranial magnetic stimulation (TMS), transcranial direct current stimulation (TDS), and deep brain stimulation (DBS). The rationale behind employing these techniques in refractory PTSD is the altered neurocircuitry seen in PTSD patients, which can be visualized on imaging. Studies involving the use of DBS for PTSD primarily target specific areas in the brain: the amygdala, the prefrontal cortex, the hippocampus, and the hypothalamus. This article aims to provide a brief overview of the various neuromodulation techniques currently employed in the management of treatment-resistant PTSD and an in-depth review of the available literature on animal models in which DBS for PTSD has been researched. We also shed light on the human clinical trials conducted for the same.

## Introduction and background

After the war in Vietnam in 1980, the first clinical documentation of post-traumatic stress disorder was seen in The Diagnostic and Statistical Manual of Mental Disorders, Third Edition (DSM-III). The increased incidence of PTSD (post-traumatic stress disorder) in war veterans was popularly referred to as "shell shock syndrome." Thus, among the various other names used to refer to post-traumatic stress disorder, "shell shock" and "war neurosis" are widely employed [1]. Then followed DSM-IV, in which the diagnostic spectrum of PTSD was expanded and PTSD was defined as an anxiety disorder with its key feature being the genesis of a few identifiable symptoms after being subjected to catastrophic stimuli. The traumatic event discussed could involve death, injury, or an insult to one's integrity. The event could either be experienced firsthand, witnessed, or learned about. People who have experienced such a profoundly traumatic event usually react with extreme anxiety, a feeling of helplessness, and terror. The hallmark symptoms include reliving the traumatic incident repeatedly, regardless of the current environment, avoiding related triggers, and showing sustained signs of elevated arousal. It is a crippling disorder that requires lifelong treatment. Typically, antidepressants and antipsychotics are prescribed in addition to psychotherapy. However, a substantial proportion of patients do not respond to treatment modalities, thus rendering them refractory. This incidence is markedly higher in defence personnel who have been through traumatic, life-altering experiences [2]. Out of all psychiatric disorders, PTSD shows the highest comorbidity rates. PTSD also has the highest rate of suicide attempts, with a 15-fold higher risk compared to other disorders [3].

Methodology

**Figure 1:**
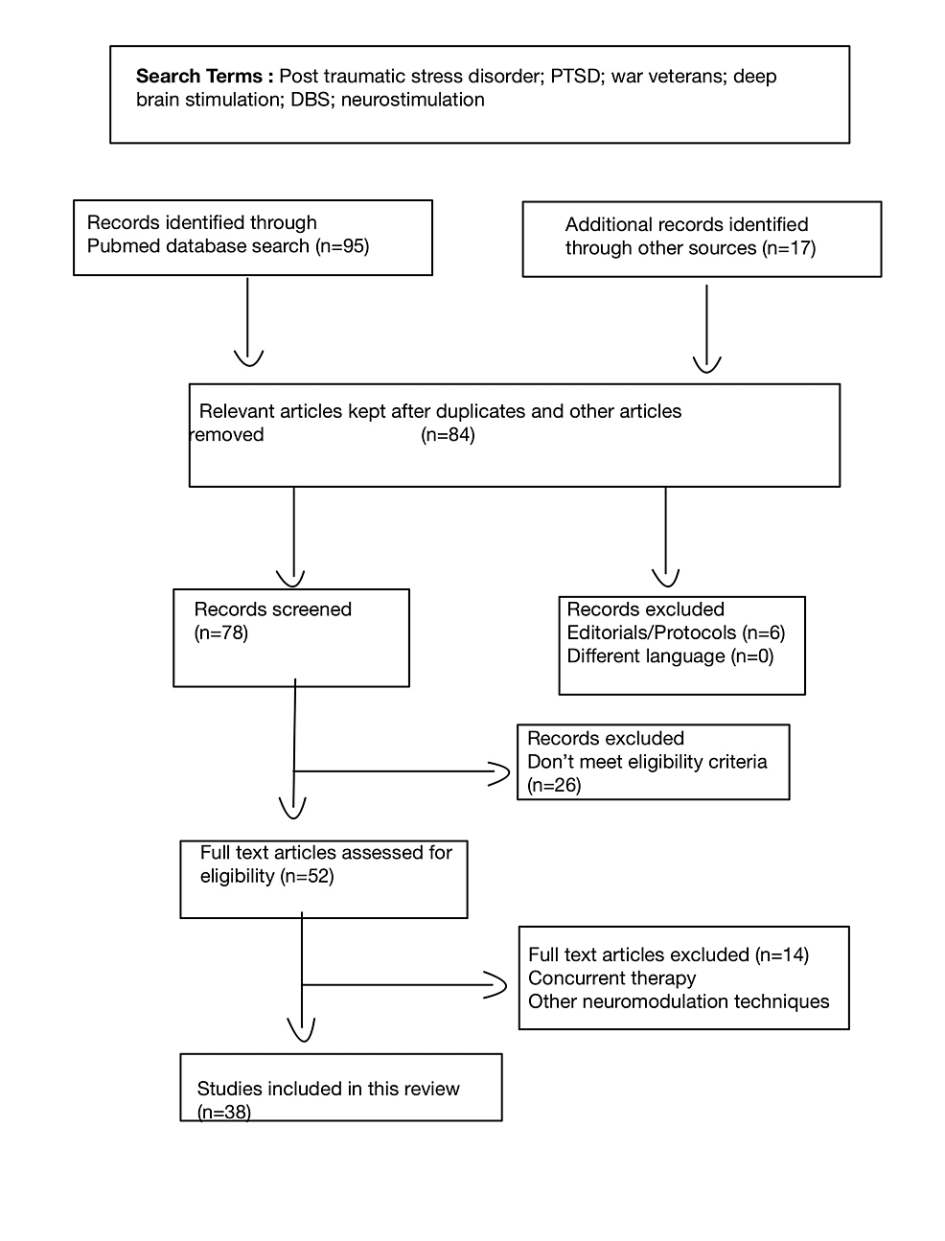
PRISMA flowchart describing record selection

An extensive search was performed on PubMed, Medline, Cochrane, and Google Scholar with the keywords "deep brain stimulation," "DBS," "neurostimulation," "PTSD," "post-traumatic stress disorder," and "war veterans." Research criteria were restricted to clinical trials and review articles with no limitation on publication date. The bibliographies of the referenced articles revealed supplementary texts, which resulted in 17 articles. Only English-language references about deep brain stimulation (DBS) and PTSD were used. A total of 36 review articles and two clinical trials were found. The Preferred Reporting Items for Systematic Reviews and Meta-Analyses (PRISMA) flowchart showcasing the record selection is indicated in Figure 1). All articles targeted only four areas: the medial prefrontal cortex (mPFC), the ventral striatum, the amygdala, and the hippocampus. Clinical trials on humans targeted the basolateral amygdala (2 publications) and the mPFC (1 publication).

PTSD

PTSD is an anxiety disorder marked by a symptom cluster that follows exposure to a traumatic event, leading to cognitive, behavioural, and physiological changes. Symptoms of the condition can be clustered into three broad categories: repeatedly reliving the traumatic experience through flashbacks, nightmares, and hallucinations; avoidance of triggering stimuli coupled with numbing of general responsiveness; and increased arousal symptoms, which include insomnia, being easily startled, being jumpy, irritability, an increased heart rate, and sweat gland hyperactivity, amongst others. It involves the development of one or more of these characteristic symptoms following exposure to a frightful, frightening, or distressing event. Symptoms should begin within six months of the traumatic event; however, in some instances, there could be a delayed onset, and the symptoms must last for a minimum of one month. People at high risk of developing PTSD include victims of sexual assault, war veterans, natural disasters, terrorist attacks, and serious accidents. Panic disorders, agoraphobia, OCD, major depressive disorder, and substance abuse disorders are more common in PTSD patients. However, it is unknown whether these comorbidities serve as a risk factor for PTSD or occur as a consequence.

Criteria for Diagnosing PTSD

According to DMS-V, PTSD has eight diagnostic criteria. Criteria A: Exposure to death (including the threat of death), grave injury, or sexual assault in one or more ways: directly experiencing the trauma, witnessing the trauma, or learning about the trauma. It also includes experiencing repetitive exposure to the traumatic event (i.e., picking up remains from the event site). Criteria B: The presence of one or more intrusive symptoms, such as recurring, involuntary, deeply disturbing recollections of the trauma, repeated distressing dreams centred on the context of the traumatic event, flashbacks, and intense distress in response to internal and external cues significant to the traumatic event. Criteria C: Continuous aversion to stimuli resembling traumatic events. The following behaviours can witness this trait: the avoidance of or desire to avoid external reminders (such as people, places, or activities) that bring up memories, thoughts, and emotions concerning the incident. Criteria D: Significant negative change in mood and cognition following the traumatic event, as evidenced by two (or more) of the following symptoms: anhedonia, forgetting important details about the incident, emotional detachment, suffering from irrational thoughts about oneself, others, or the world, misconceptions about what happened and why it happened, and persistently negative emotional feelings. Criteria E: Significant changes in arousal and reactivity begin shortly after the trauma or are worsened by the trauma. Signs of increased arousal (must have two or more): uncontrollable rages and irritable outbursts; self-inflicted harm or recklessness; hypervigilance; exaggerated arousal; difficulty paying attention; and disruption of sleep. Criteria F: The duration of criteria B, C, D, and E should be more than 30 days. Criteria G: A perturbation produces clinically considerable suffering or impairment that interferes with cognitive, occupational, and social aspects of functioning. Criteria H: This disturbance cannot be attributed to a substance, drug, or concurrent medical condition. Based on these criteria, a clinically administered PTSD scale (CAPS score) is used to determine the degree of the disorder.

Physiological Neural Pathways of Fear Conditioning and Extinction 

The neural pathway of fear conditioning involves the amygdala, the prefrontal cortex, and the hippocampus. Since the amygdala plays a key role in transmitting various emotions, especially fear, it is of huge importance. Sensory information transmitted through the thalamo-amygdala pathway reaches the amygdala more rapidly than sensory information transmitted from the cortex to the amygdala. The thalamic sensory nuclei and the primary sensory centres of the cerebral cortex supply the amygdala with sensory afferent fibres. The basal nucleus (BA), lateral nucleus (LA), and accessory basal nucleus are all grouped in this complex, which is the principal recipient of these afferent fibres [4]. Lesions to the basolateral region eliminate traditionally established fear conditioning. The basolateral amygdala contains the nuclei mentioned above: the central nucleus (CE), intercalated cell clusters (ITC), and the cortical nucleus. The central nucleus receives "danger signals" from the LA through somatic, visual, and auditory sensory fibres. Connectivity between the CE and other parts of the brainstem involved in generating the distinct autonomic signals of fear is extensive [4]. Each structure mentioned above is known as the "wheelhouse" for a particular thing. While the amygdala is key for extinction learning, the ventromedial prefrontal cortex (vmPFC) is largely responsible for suppressing the conditioned fear response during extinction recall [5]. This function is partly due to the neural plasticity of the vmPFC. The amygdala's primary checkpoint, the CE, receives the inhibitory signals sent from the BLa to the ITC neurons. It is due to this reason that these neurons are also termed "mediators of fear extinction" [6]. The CE contains only GABAergic neurons and is responsible for transmitting signals to the hypothalamus and brainstem [7]. There are multiple neural pathways seen in fear conditioning, extinction, and PTSD. These dysfunctional neural pathways involving the amygdala, the prefrontal regions, and the hippocampus lead to the conflation of fear memories and the persistence of the learned fear responses [8].

Etiopathogenesis of PTSD

Recent studies have discovered that a hyperactive amygdala and dorsal anterior cingulate cortex can predispose a person to develop PTSD [9,10]. Other risk factors include genetic variances, hormonal imbalances, a decline in neurotrophic factors, and aberrant monoamine and neuropeptide concentrations in the bloodstream.

Neurocircuitry of PTSD

PTSD is still a complex disease, and many models have been made to understand why and how it occurs. One such model is "threat sensitivity," which suggests that PTSD symptoms arise from a failure to inhibit a maladaptive fear response after the traumatic event. A deeply traumatic event such as war or rape leads to an unshakeable, conditioned fear response. This response leads the patient to associate the stimulus present in that traumatic event with an aversive event. This conditioned fear response can be suppressed or overridden by extinction learning. Extinction learning aims to make the patient associate the same stimulus with the ability to prevent or manage an aversive event in a safe environment. Extinction learning, therefore, forms the crux of exposure therapy.

Structural Changes in PTSD

**Figure 2:**
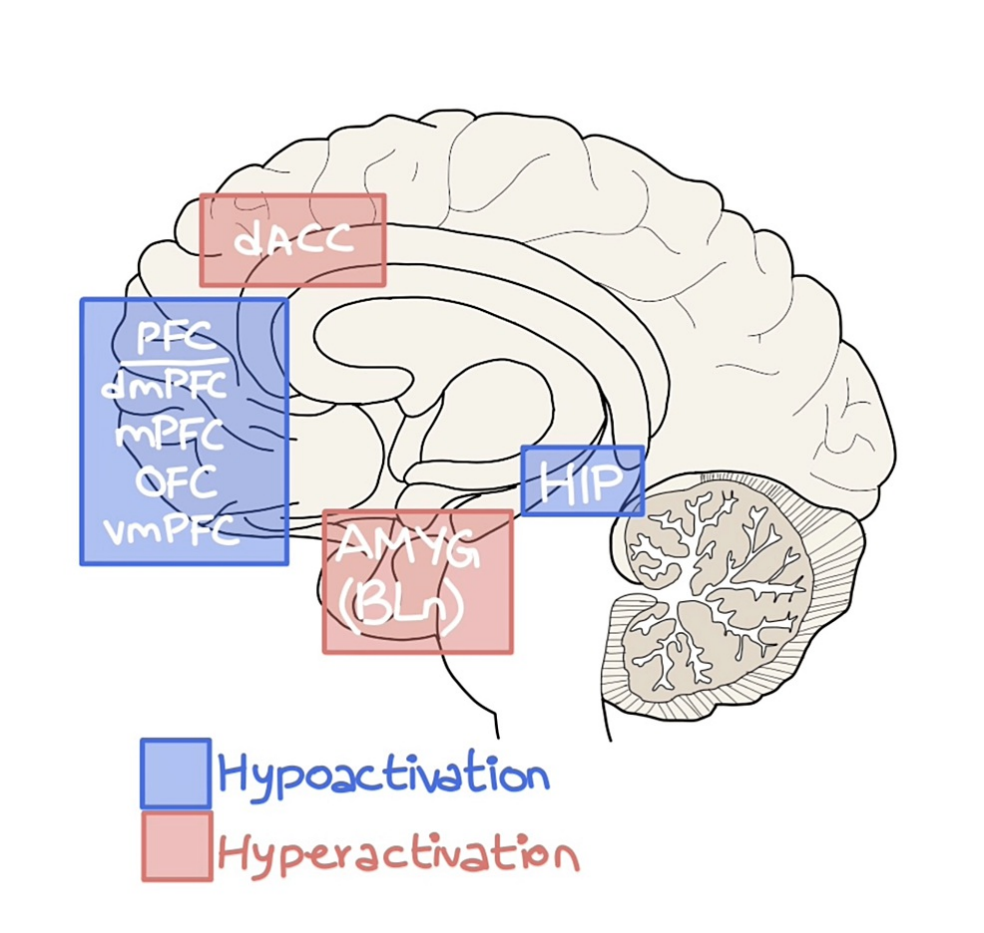
The hyperactive (red) and hypoactive (blue) areas of the brain in PTSD dACC: dorsal anterior cingulate cortex; dmPFC: dorsomedial prefrontal cortex; mPFC: medial prefrontal cortex; OFC: orbitofrontal cortex; vmPFC: ventromedial prefrontal cortex; AMYG: amygdala; BLn: basolateral nucleus of the amygdala; HIP: hippocampus

Imaging studies such as MRI, functional magnetic resonance imaging (fMRI), and PET scans revealed anomalies in certain brain areas that may play a role in the development or origin of PTSD. These aberrations predominantly lie in the amygdala, the medial prefrontal cortex (mPFC), the hippocampus (HPC), the anterior cingulate cortex (ACC), and the ventral striatum (VS). Imaging studies noticed changes during both active recall and rest. As a result, blood flow to the amygdala rose, whereas, in the superior frontal gyrus, the parietal and temporal areas decreased (as shown in Figure 2) [11].

Conventional Treatment

The traditional FDA-approved treatment of PTSD is all-encompassing and consists of pharmacotherapy such as selective serotonin reuptake inhibitors (SSRIs) and psychotherapy, which entails cognitive behavioural therapy, exposure therapy, and eye movement desensitisation and reprocessing (EMDR), which is a recent addition. The most studied therapy modalities commonly employed in PTSD treatment are trauma-focused cognitive behavioural therapy (TFCBT) and EMDR [12]. Commonly employed drugs in PTSD are sertraline, paroxetine, fluoxetine, risperidone, topiramate, and venlafaxine. Most of these drugs are either SSRIs, serotonin and norepinephrine reuptake inhibitors (SNRIs), or antipsychotics. Coupling such drugs with psychotherapy is the treatment of choice. Nonetheless, many patients are only partially or completely resistant to medical treatment and are diagnosed with refractory PTSD. The duration after which a patient can be considered resistant to treatment is poorly defined, and the effect of comorbidities such as substance abuse on the refractoriness of PTSD is also not well known.

Exposure Therapy

Exposure therapy is commonly employed in managing PTSD; it consists of repeated exposure to the traumatic stressor without the experience of the anticipated and deeply feared consequences. These new memories based on extinction learning can also be termed "safety memories." The ability to recall the learned safety memories is known as "extinction retention." If the safety memories are duly recalled, they suppress the initially learned fear responses and work towards keeping the symptoms of PTSD at bay. Then again, a high number of abnormalities in extinction retention are seen in PTSD; this is thought to be why most PTSD patients experience a symptomatic relapse at least once in their lives. Furthermore, abnormalities can also be noticed in extinction learning, which explains why some patients experience symptoms even after years of exposure therapy. Thus, it is imperative in the treatment of PTSD that exposure therapy is conducted in different contexts and with different stimuli.

Neuromodulation

Neuromodulation refers to the alteration or modulation of nerve activity by delivering electrical or pharmaceutical agents directly to a target area. Neuromodulation therapies include electroconvulsive therapy (ECT), transcranial magnetic stimulation, transcranial direct stimulation, vagus nerve stimulation, trigeminal nerve stimulation, and deep brain stimulation. Out of these, ECT is the oldest recorded form of neuromodulation. Neuromodulation therapies are highly targeted to specific areas in the brain or the spinal cord, as opposed to being systemic, i.e., pharmaceutical treatments. This allows them to avoid the side effects commonly associated with systemic treatment. These techniques are also easily reversible, which provides a great deal of therapeutic control to the patient and the physician, as the stimulus parameters can be adjusted or ceased at any time. In contrast to therapies that call for intermittent dosing, these treatments have a high rate of compliance. These therapies can be of two types: invasive and non-invasive.

Electroconvulsive Therapy

Electroconvulsive therapy (ECT) is the oldest form of neuromodulation. It involves the repeated administration of current to the brain under general anaesthesia with the aim of inducing a generalised tonic-clonic seizure. It is analogous to medication and psychotherapy in that it targets brain circuits in a non-selective manner [13]. This leads to a number of systemic side effects, making this method less preferable over the newer ones. Repetitive TMS (rTMS) leads to long-term modifications in the targeted underlying circuit.

Transcranial Magnetic Stimulation

Transcranial Magnetic Stimulation (TMS) involves the placement of a coil containing a magnet over a portion of the patient's scalp. Rapidly fluctuating electrical current is passed through the coils, which creates a magnetic field. This time-varying magnetic field induces an electrical current in the cortex. This technique is safe and usually well tolerated [14], with the most common side effect being headaches. Seizures are extremely unlikely. 

Transcranial Direct Stimulation

In this technique, a low, non-convulsive current is applied non-invasively to the brain via an anode and the current returns via a cathode. This constant current affects the excitability of the cortex by depolarizing (via the anode) or hyperpolarizing (via the cathode) the neurons. This technique is conducted on awake, conscious patients and the side effects commonly reported are headache or target-site irritation. As of now, the FDA has not approved the use of TDS for any clinical indication [13].

Vagus Nerve Stimulation

A bipolar electrode conducting low-frequency electrical pulses is wrapped around the cervical vagus nerve. The electrode is connected to a pulse generator, which is implanted subcutaneously in the anterior chest wall. Vagus nerve stimulation is employed in the treatment of refractory epilepsy and is used as adjunctive therapy in the treatment of refractory depression [15]. Trigeminal nerve stimulation works on the same principle as vagus nerve stimulation and acts on the forebrain circuitry.

Deep brain stimulation

Deep brain stimulation (DBS) is a form of invasive neuromodulation that uses stereotactic bilateral or unilateral implantation of platinum-iridium electrodes, which measure 1.3 mm in diameter, into a targeted brain area. With several stimulating attachments at the electrode tip, it can accurately target specific brain regions [13]. A connecting wire establishes a connection between the electrodes and a pulse generator. The pulse generator is implanted subcutaneously. either subclavicularly or abdominally, and with the help of a hand-held wireless programmer, the stimulus settings may be continually and easily modified without invasive surgery. An area within the range of a millimetre is frequently chosen for stimulation [16]. High-frequency stimulation (stimulation in the range of 130 Hz) is used to treat a hyperactive structure such as the amygdala in PTSD. Deep brain stimulation targets the deeper structures in the brain, such as the amygdala, which cannot be reached by TMS or TDS. The biggest drawback of this technique is the "power issue" [17]. The batteries of the pulse generator need to be changed every two to three years, for which surgery is necessary.

Open-Loop and Closed-Loop DBS

There are two modalities of deep brain stimulation available: open-loop (also known as conventional) and closed-loop (known as adaptive). In the aforementioned open-loop DBS, a pulse generator is implanted subcutaneously, and the stimulus parameters such as frequency, amplitude, and duration are pre-set and remain constant. Thus, in open-loop DBS, the consulting neurologist must manually change the parameters every three to 12 months based on the patient's condition and treatment results [18]. Closed-loop DBS employs the use of biomarkers, which enables it to receive constant biofeedback from the patient's brain and/or body and readjust the parameters accordingly. When the brain is in a dysfunctional state, stimulation pulses are released, or they are automatically and dynamically modified based on fluctuations in the recorded signal over time [19]. In the case of PTSD, these biomarkers could be the skin conductance rate [20] or the hypoactive prefrontal cortex, which could lead to a change in the stimulation of the hyperactive amygdala (where the electrodes would be implanted).

Uses in Psychiatry

Since DBS is one of the most invasive newer neuromodulation procedures, it is only opted for in the management of treatment-refractory cases such as treatment-refractory OCD and treatment-refractory epilepsy. Other FDA-approved clinical indications of DBS are in managing neurodegenerative movement disorders such as Parkinson's disease, dystonia, and essential tremors, in which DBS has shown promising results. Even in various psychiatric disorders such as depression, Tourette's syndrome, and others, DBS has shown commendable outcomes. For this reason, the application of DBS to PTSD is being pursued in depth. DBS is a relatively safe treatment; the side effect profile of this method is low with the common side effects being mood changes, peri-operative headaches and wound infection [21]. Suicidal ideation is a rare complaint among those undergoing this treatment and is commonly seen when DBS is being used to treat treatment-resistant depression. Sometimes, patients also complain of limitations in neck movements if the pulse generator is implanted below the clavicle.

Deep Brain Stimulation in PTSD

At the very base of it, PTSD is fundamentally a problem of neural circuits. The studies involving the application of DBS in the treatment of refractory PTSD primarily targeted four areas of the brain: the amygdala, the prefrontal cortex, the hippocampus, and the ventral striatum. With the help of imaging studies, it has been found that the amygdala, particularly the basolateral nucleus of the amygdala, is hyperactive in patients with PTSD which leads to the symptoms of hyperarousal and an exaggerated fear response. It has been postulated that high-frequency stimulation of the BLa could help suppress these symptoms [22]. Furthermore, stimulation of the hypoactive prefrontal cortex alleviates fear responses and anxiety-like symptoms and regulates the hyperactive BLa seen in PTSD patients [23]. The aim of various studies employing methods of neurostimulation is to re-establish equilibrium between the hyperactive structures (the amygdala and the anterior cingulate cortex) and the hypoactive structures (the hippocampus and the prefrontal cortex) and to achieve long-lasting homeostasis [24].

## Review

In the last few decades, a great deal of research has been done with the help of neuroimaging studies, which has given us great insight into the contribution of different brain areas concerning emotional and cognitive functions in both the healthy and ill brain [25]. The commonly researched target areas include the basolateral amygdala, the ventral striatum, the hippocampus, and the prefrontal cortex. Research (as of July 2022) has only been conducted on rats and humans.

PTSD in rat models

A total of 24 publications were found detailing the results of deep brain stimulation and PTSD in rats. All publications were based on fear conditioning and extinction paradigms. Research studies have primarily focused on four areas: the prefrontal cortex, the hippocampus, the ventral striatum, and the basolateral amygdala.

Prefrontal Cortex

The prefrontal cortex is responsible for the inhibition of the amygdala and is commonly found to be hypoactive in PTSD. The prefrontal cortex is divided into the prelimbic and infralimbic regions. Stimulation applied to the prelimbic region during extinction increased freezing behaviour during extinction recall, whereas stimulation applied to the interleukin (IL) region decreased freezing behaviour, indicating an anxiolytic effect-a result also found by others [23,26].

Hippocampus

The hippocampus is a key research area in PTSD due to its importance in the stress response and corticotropin release. There is pathological hippocampus degeneration in PTSD, along with a smaller hippocampal volume. Numerous studies involving DBS and targeting the hippocampus revealed that behavioural improvements depend on the target region inside the hippocampus and the concurrent stimulation protocols. While high frequency reduces freezing behaviour during recall sessions, the outcomes of low-frequency stimulation depend on the area targeted [27,28]. Low-frequency stimulation (2 Hz) when applied to dorsal Cornu Ammonis 1 and 2 regions resulted in increased freezing during recall, whereas low-frequency stimulation applied to the ventral aspect of Cornu Ammonis 1 resulted in decreased freezing behaviour during extinction [29-31].

Ventral Striatum

Electrodes were implanted in the ventral striatum, dorsal and ventral to the anterior commissure. Stimulation of electrodes placed dorsally decreases freezing behaviour, whereas stimulation of electrodes placed ventral to the AC leads to an increase in freezing behaviour. Electrodes placed in the core VS only showed reduced freezing behaviour during extinction retrieval [32-35].

Amygdala

The most highlighted target area in PTSD is the amygdala, specifically the basolateral amygdala. Due to the amygdala being hyperactive in PTSD, high-frequency stimulation is commonly employed to decrease its activity. This procedure has been shown to alleviate the symptoms of PTSD significantly. Increased cerebral perfusion is seen in the BLa. The PFC indirectly controls the activity of the amygdala. All research involving the basolateral amygdala is based on the "defensive burying" model. In this, it is hypothesised that it is the innate behaviour of the rat to bury objects that are viewed as threatening or dangerous, and treatments that work on reducing the animal's tendency to bury such objects are anxiolytic. Through the work of Saldivar-Gonzalez et al., Sui et al., Langevin et al., and Stidd et al., it was found that the animals subjected to DBS in the right BLa amygdala showed decreased burying, spent less time burying painful stimuli, and it led to a decrease in freezing behaviour following fear conditioning [36-38].

PTSD in human models

While PTSD has been extensively researched in rats, only six publications detailing the treatment of three patients were found. Two of the three patients were combat veterans, and the other was a victim of domestic abuse. All patients showed resistance to conventional treatments. The first patient was a combat veteran who had undergone 20 years of pharmacotherapy and psychotherapy but remained severely symptomatic. According to the diagnostic criteria, his CAPS (clinically administered PTSD scale) score was 119, labelling him with severe PTSD. With the help of imaging studies, higher amygdala metabolism was found when a patient was asked to recall the traumatic event compared to metabolism at rest. Electrodes were surgically implanted in the basolateral amygdala on both the right and left sides. High-frequency stimulation (~160Hz) was given, following which progress was tracked. There was marked improvement at the eight-month post-op mark, and the CAPS score had decreased to 72. The CAPS score later became 62, fifteen months postoperatively [39-41]. The patient reported the cessation of crippling nightmares for a year. However, the patient was hospitalised at 17 months post-treatment due to suicidal ideation [42]. This fact emphasises the high chance of relapsing into symptoms of PTSD and the need for constant therapy and monitoring. The second patient is a 40-year-old male who is also a war veteran. The patient was given high-frequency stimulation at 130 Hz in the BLa. All other stimulus parameters were kept the same as for the first patient. Seven months post-surgery, a decrease of more than 30% of the original CAPS score was noted [13]. A marked reduction in anger dyscontrol was seen, which allowed the patient to partake in the social activities he had earlier avoided. The third patient is a woman in her late 40s who suffered as a victim of domestic, physical, sexual, and verbal abuse for 17 years. In this case, the target areas of the brain were the mPFC and the uncinate fasciculus. The initial CAPS score of the patient was 56. DBS electrodes were implanted in the target region, centred on the subgenual cingulum, and showed maximum contact with the uncinate fasciculus. Electrostimulation was done at 130 Hz. Post-treatment, the patient could tolerate triggers that reminded her of the past trauma without any adverse symptoms. Seven months postoperatively, the CAPS score was 0 (100% improvement), and the patient could return to her daily life and enjoy the same social activities and responsibilities as she used to do before the onset of PTSD [43].

Prospective application

PTSD is one of the only psychiatric disorders that has undergone extensive animal research before human clinical trials. The rationale behind DBS for PTSD has been widely accepted, as evidenced by the publications on rat models. In the rat models, the target areas of the brain were those involved in fear circuitry, the extinction learning pathway, and the pathways responsible for the behavioural reaction to conditioned fear. These areas were the amygdala (in particular the BLa region), the PFC (especially the vmPFC), the ventral striatum, and the hippocampus. However, in humans, as of July 2022, DBS has only been tried in 3 patients with PTSD worldwide. The DBS targets in these cases were the BLa and the mPFC. Thus, the scope of DBS in other target areas such as the hippocampus and the VS is fruitful. Studies have found a smaller hippocampal volume alongside hippocampal degeneration in PTSD. The use of DBS to ameliorate the symptoms caused by structural changes in the hippocampus must be researched shortly, as it could be of immense importance. PTSD is a highly heterogeneous illness that can be supplemented with particular psychiatric diagnoses; thus, defining unique, customised treatment for this patient population can prove to be a task [44].

DBS with psychotherapy

PTSD is a complex disorder with a unique cocktail of symptoms in each individual. The differences in the trauma experienced can drastically affect how each person presents with the disorder. A theranostic approach should be adopted when a patient first walks in the door. This approach involves coupling conventionally used treatments such as pharmacotherapy and psychotherapy but ultimately resorting to neuromodulation procedures such as DBS if refractoriness is noted. DBS or other neuromodulation techniques, if used in conjunction with exposure therapy, could provide favourable outcomes and a decreased risk of symptomatic relapse.

## Conclusions

Refractory PTSD is an important mental health issue, with approximately one-third of the total cases being resistant to treatment. Cases of PTSD have increased dramatically in recent years, owing primarily to the pandemic. Thus, refractory PTSD is a big problem whose treatment must be looked into. Multiple neuromodulation techniques are being looked into for this purpose, including DBS. Other novel techniques, such as eye movement desensitisation and reprocessing (EMDR), have started being adopted for treating this disorder too, but these techniques, too, have a long way to go. As of now, the amygdala and prefrontal cortex are the only places where DBS can be used to alleviate the symptoms of PTSD. However, thorough research has been conducted on rats, which provided us with a clue into the altered neural circuitry in PTSD. All publications on animals and humans have shown promising results. However, DBS for PTSD remains highly investigational and requires much research to become a routine treatment protocol.
